# Alcohol health warnings can influence the speed of consumption

**DOI:** 10.1007/s10389-016-0770-3

**Published:** 2016-10-26

**Authors:** Lorenzo D. Stafford, Joe Salmon

**Affiliations:** Department of Psychology, University of Portsmouth, King Henry Building, King Henry I Street, Portsmouth, PO1 2DY UK

**Keywords:** Alcohol health warning, Alcohol consumption, Alcohol information

## Abstract

**Aim:**

Recent research has shown that adopting strong (i.e. high fear) visual health-warning messages can increase the perceived health risks and intentions to reduce alcohol consumption. Separately, it is known that the speed at which alcohol is consumed has dramatic effects on the level of intoxication. In the present study we aimed to combine these two separate areas to understand whether the speed of alcohol consumption is influenced by the type of alcohol health warning contained on the beverage.

**Subject and methods:**

In the present study, female participants (*N* = 45) consumed an alcoholic beverage in a relaxed environment in one of three conditions: no health-warning label, a text-only health-warning label or a pictorial health-warning label with text.

**Results:**

We found that compared with the control condition, the beverage was consumed at a slower rate in the two health-warning conditions, which surprisingly did not differ from each other. Despite these effects, product acceptability did not differ between the text-only and control conditions.

**Conclusion:**

These are the first set of results to demonstrate how the use of strong health warnings on alcoholic beverages can influence actual drinking rate and further suggest that the beneficial effects of slowed consumption are possible in the absence of any reduction in consumer acceptability.

## Background

Alcohol is one of the most widely consumed drugs, integrated into the fabric of many cultures around the world; but this relationship comes at a price. In the UK alone, it is believed that 9 million adults drink to a level that poses health risks, with over 1.5 million individuals having some degree of alcohol dependence (Public Health 2014). This does not just impact the individual, it has bearings on society, and economics; alcohol-related harm is thought to have cost £21 billion in 2013–2014, with £3.5 billion of that cost related to alcohol issues treated via the National Health Service (NHS; Public Health 2014). Given these statistics, it is therefore imperative that strategies for curbing the excessive consumption of alcohol are considered and research engaged to examine their effectiveness. As part of this process, it is worthwhile learning any lessons from the huge change in public attitudes and behaviour to another widely consumed drug: Nicotine. In England, smoking has decreased from 39 % of adults in 1980 to 20 % in 2010 (HSCIC 2013). The factors responsible for this significant change are a combination that include health-warning labels on packaging, education on the damaging effects of smoking and smoke free legislation (Martin-Moreno et al. 2013). In terms of warning labels, research has shown that they are an important reminder on the danger of smoking and are strongly linked to intentions to stop smoking (Hammond 2006).

The effectiveness of health-warning labels in relation to tobacco have been examined with several factors being identified. Large warning labels are most effective, being large enough to be easily noticed and read (Hammond et al. 2007); Smokers report greater recall for warning labels on the front of packages, and that if the label had a direct and accurate message in conjunction with being simple, a greater impact is found (Hammond 2011); pictorial health-warning labels are more effective than text-only as pictures increase the accessibility to a greater target audience, whilst allowing smokers to visualise problematic scenarios (Hammond et al. 2004); also, colour pictures are more effective than black and white pictures.

In terms of alcohol, research has investigated public acceptability and consumer aspects of alcohol warning messages (e.g. Jarvis and Pettigrew 2013; Martin-Moreno et al. 2013; Thomson et al. 2012), but very little work has examined whether such warning messages can actually change attitudes and virtually none looking at behavioural change. One Internet-based study found that when confronted with choices of which beverage to select with warning messages, young drinkers showed a reduction in choosing the alcoholic beverage (Jarvis and Pettigrew 2013). Work has also demonstrated that advertisements with fear arousing alcohol related visuals (e.g. ambulance at crash scene caused by excess alcohol) led to increases in perceived risks associated to alcohol use (Slater et al. 2002). Theoretically, those findings supported the Extended Parallel Processing Model (EPPM, De Hoog et al. 2007) which predicts that messages accompanied by threatening visuals induce more processing of the accompanying message. Most recently, we conducted an experiment (Wigg and Stafford 2016) where individuals were exposed to alcoholic beverages in three separate conditions (no health warning, text-only, pictorial), and found that participants exposed to the pictorial condition had higher intentions to reduce and quit alcohol consumption; which was related to individuals level of fear arousal.

The aforementioned study provided a useful first step in establishing experimental protocols in this domain; however, it was unclear from that work alone if such health-warning labels can influence other behavioural measures related to alcohol consumption. One such measure is the speed at which alcohol is consumed, which is important since the same quantity of alcohol drunk in a short versus longer period of time will have dramatically different effects and thereby risks associated to individual health and safety (Moskowitz and Burns 1976; Bernosky‐Smith et al. 2012). Episodic drinking rate is driven by a number of factors including the sensory (taste) aspects of the drink that cue the presence of alcohol strength and the perceived behavioural (intoxicating) effects of alcohol (Higgs et al. 2008). It would also seem probable that speed of consumption is related to ‘protective drinking behaviours’ (Ray et al. 2009); the extent to which individuals engage in practices such as pacing the number of drinks consumed in a time period. Work has shown that individuals who score higher in protective drinking behaviours also have fewer negative consequences of alcohol consumption (Ray et al. 2009), which reinforces the point regarding the importance of the rate at which alcohol is consumed.

The current study therefore aimed to examine whether health-warning messages have the ability to influence this important aspect of alcohol behaviour. To test this question, individuals consumed the same alcoholic beverage in one of three separate conditions that varied in the type of health-warning on the bottle: no health-warning label; text-only; pictorial and text health-warning label. We tentatively predict that consumption rate will be fastest in the ‘no health-warning label’ category compared to both text-only and pictorial conditions, with the latter predicted to have the slowest rate.

## Methods

### Participants

Forty-five female university students, aged between 18 and 25 years (*M* = 18.93, *SD* = 1.12) participated in this study. Participants were recruited using an online system and via social media with the study advertised as ‘factors involved in alcohol related behaviour’. Participants were invited to take part only if they were aged between 18 and 25 years, were female and regular consumers of alcohol (see AUQ). All participants were in good health and to our knowledge free of any somatic diseases. The study protocol was given ethical approval from the department’s ethics committee (British Psychology Society guidelines, similar to the Declaration of Helsinki).

### Design

A between-subjects design was used, where participants were randomly allocated to one of three groups based on the health-warning label condition: control (no health-warning label), text only health-warning label, or pictorial health-warning label with text (Table 1). The duration to consume the test beverage was the main dependent variable.

**Table 1:** Mean (SEM) participant characteristics

	Group						
	Control		Text only		Pictorial		Group differences
	M	SE	M	SE	M	SE	
Age	18.9	0.3	18.8	0.3	19.1	0.2	*F* = 0.36, NS
UK alcohol units (p/week)	16.1	2.4	15.5	2.8	12.9	1.4	*F* = 0.56, NS
MAST	0.8	0.2	0.3	0.1	0.7	0.2	*F* = 2.14, NS
PB-total	8.7	0.4	9.2	0.5	2.1	0.5	*F* = 0.35, NS
PB-pacing	10.1	0.6	11.5	0.7	9.5	0.9	*F* = 1.96, NS
PB-setting limits	7.8	0.9	8.1	1.1	9.5	1.3	*F* = 0.69, NS
PB-social	10.8	0.3	10.3	0.4	10.7	0.2	*F* = 0.78, NS
PB-diluting	6.0	0.9	7.0	0.8	6.3	0.7	*F* = 0.35, NS
No. of smokers	1		1		0		

### Materials

#### Alcohol usage questionnaire (AUQ)

Habitual alcohol consumption patterns were measured using a questionnaire (based upon Mehrebian and Russell 1978). Participants were accepted into the study only if their total weekly alcohol consumption was between 2 and 40 units of alcohol, consistent with previous research (Higgs et al. 2008; Stafford and Dodd 2013). Additionally, since the test beverage was vodka based, it was mandatory that participant’s habitual alcohol consumption included vodka-based beverages to avoid compromising the validity of the study.

#### Protective behaviours

The extent to which individuals used protective behaviours whilst drinking alcohol was measured using the Protective Behaviours questionnaire (Ray et al. 2009). The questionnaire comprises 16 questions, e.g. ‘I pace my drinks to 1 or fewer per hour’. Participants respond using a five point Likert scale ranging from *Always true* to *Never true*. The questionnaire yields scores in four different categories: pacing, setting limits, social, diluting.

#### Michigan alcohol screening test (MAST)

Participants were required to complete the MAST test before participating in the study. In order to avoid using any individuals with existing or potential alcohol related problems, individuals were only included in the study if they scored *No apparent problem* (0–2) on the 22 question test.

#### Health-warning label

The health-warning labels were based on Wigg and Stafford’s (2016) design. The text-only health-warning label comprised of the statement ‘Alcohol causes fatal liver cancer’. The same text was used for the pictorial health-warning label with an image of a diseased liver. The health-warning labels were placed on the front of the alcoholic beverage as Hammond (2011) found that for smokers, greater recall was present when labels were on the front of packages.

#### Product design questionnaire

Before the study began, participants had to complete a pre-test product design questionnaire of the test beverage. This consisted of five questions on the acceptability of the bottle used (see Appendix). This was to ensure that participants actively observed the bottle and the health-warning label, if present. Responses were recorded on a seven-point Likert scale, with 1 indicating the highest negativity and 7 referring to the most positivity towards the question.

#### DVD programme

As with previous similar research (Higgs et al. 2008; Stafford and Dodd 2013), in order to provide a more naturalistic environment and divert attention away from the real aim of the study, participants watched DVDs: *Planet Earth*, *Jungles* (Fothergill 2006, *Planet Earth*,* Jungles*, BBCDVD). The documentary was chosen as it is relatively neutral in tone and unlikely to induce different mood states, which may have affected the study.

#### Blood alcohol level (BAL) readings

Blood alcohol level readings were taken pre-test and post-study via a breathalyser (Alcosens DA5000 digital). Participants could only partake in the study if their initial reading was a score of 0. This reading was taken again post-study to determine if participants were safe to leave the administration room.

#### Drinks and administration

A pilot study was conducted to find the most suitable beverage to use in the study. Participants (*n* = 10) completed a questionnaire on the level of consumption and preference for a number of popular beverages. The drink with the highest ratings was ‘WKD’ (4 % ABV) and therefore used in the study. For the actual study, participants were given a 275-ml bottle of freshly opened WKD served at fridge temperature.

#### Procedure

All testing took place between 11.00 a.m. and 5.00 p.m. in test rooms at the psychology department of the university. Upon arrival, participants provided informed consent, completed a breathalyser test using a digital personal breathalyser (all readings scored zero) and then completed the MAST, AUQ, and Prevention methods questionnaire. Next, the participants completed the pre-test product design questionnaire. Participants were then instructed to consume all of the drink while watching the DVD in their own time. The duration to consume the beverage was covertly timed by the experimenter using a mobile phone; with the timing commencing when participants took their first sip and stopped when they put the empty bottle back on the table. Participants then completed a post-test product design questionnaire to ensure participants were able to recall the health-warning information (if present). Their BAL was again measured. Each participant was given a partial debrief on the purpose of the study, and told that a full debrief would be sent out by email once all participants completed the study. This was felt necessary because a full debrief may have compromised the study for future participants. For safety reasons, participants were asked to wait in a waiting area for a period of time to minimise any adverse effects of the beverage, and informed not to operate heavy machinery or drive a vehicle for at least 3 h. Course credit was administered for participation.

#### Analysis

The alcohol consumption rate was analysed using a univariate ANOVA, with the between-subjects factor of the group (no health-warning label/text only health-warning label/pictorial health-warning label with test). For the five product design questions, we completed reliability analyses and removed one item (‘feel’) with relatively low inter-item correlation, which resulted in a high Cronbachs alpha (*α* = 0.76). The remaining four questions were summed together (termed ‘acceptability’) and analysed using a univariate ANOVA, with the between-subjects factor of group (no health-warning label/text only health-warning label/pictorial health-warning label with test). Subsequent post-hoc tests were completed with Bonferroni adjustments.

## Results

### Consumption duration

**Fig. 1:**
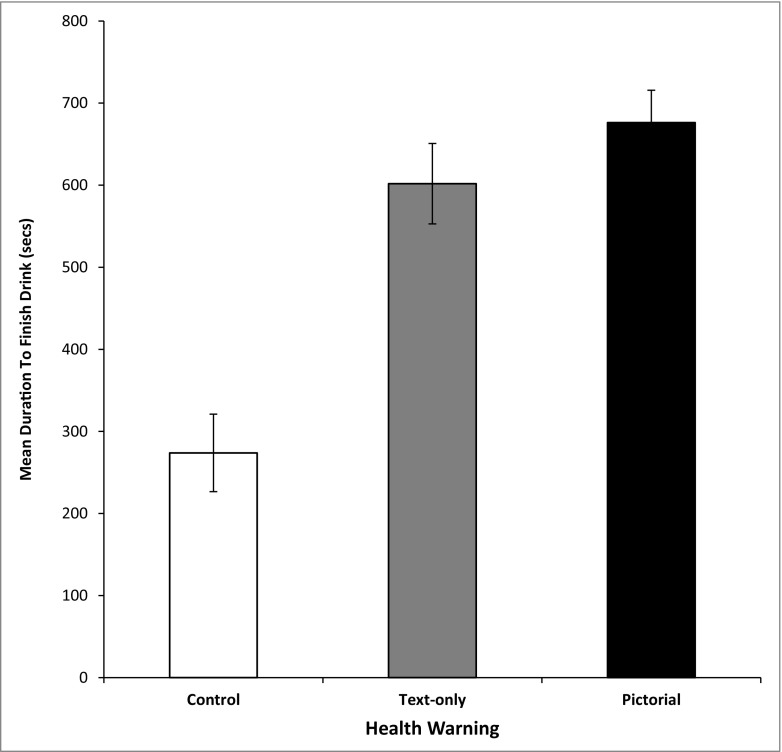
Mean duration of drinking as a function of health-warning label

Analysis revealed a significant effect of health-warning label, *F*(2, 42) = 22.25, *p* < .001,* η*_p_^2^ = .514, where consumption was significantly faster in the ‘no health warning’ compared to both the text and pictorial conditions (*p* < .001), with the latter two not differing significantly (*p* = .76; Fig. 1). This suggests that the presence of a health-warning label is more important in influencing the rate of consumption than the type of message (text, pictorial).

### Acceptability of the alcohol beverage

**Fig. 2:**
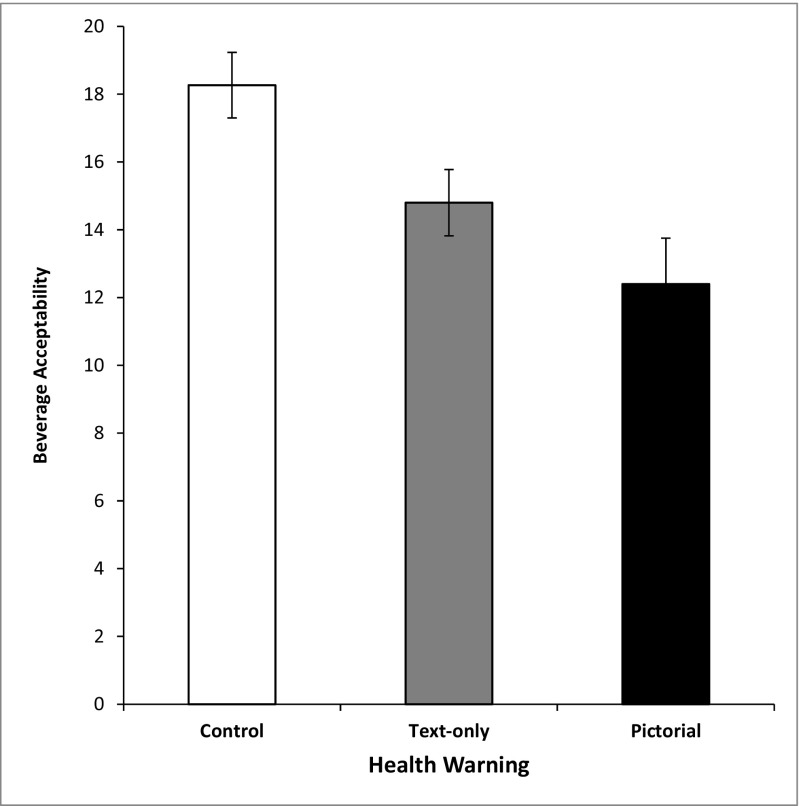
Total acceptability ratings as a function of health-warning label

A significant effect of health-warning label was found, *F*(2, 42) = 7.01, *p* = .002,* η*_p_^2^ = .25, with as expected, significantly lower ratings in the pictorial condition versus the ‘no health warning’ condition (*p* = .002), but more surprisingly, there was no difference between the latter and the text condition (*p* = .10). There were also no differences between the text and pictorial conditions (*p* = .41). This suggests that although health-warning text can yield a decrease in the speed of consumption, there is no corresponding decline in the acceptability of the alcohol beverage (Fig. 2).

### Correlations

In order to explore further the influences of the consumption rate, we computed correlations for the two health-warning label conditions, between the duration to finish the drink and protective behaviours, product acceptability scores. None of these analyses were significant (Table 2).

**Table 2:** Correlations between consumption speed, protecting behaviours and product acceptability (text-only, pictorial conditions, *N* = 30)

Measure	Correlation
Protective behaviours: pacing	0.01
Protective behaviours: setting limits	0.01
Protective behaviours: social	−0.28
Protective behaviours: diluting	0.18
Acceptability	−0.09

## Discussion

The main finding of the study was that alcohol was consumed at a faster rate for those in the control condition compared to both the pictorial health-warning label and text only conditions. This pattern of findings is generally consistent with prediction and extends previous research (Wigg and Stafford 2016) to demonstrate that health-warning labels can also influence episodic drinking behaviour. The mechanism responsible for slower consumption is theorised to be due to higher levels of fear arousal in the two health-warning conditions. It is well established that health-warning messages that elicit fear can be an effective means of informing the public and also instrumental in intentions to quit smoking (Kees et al. 2010). For alcohol, it is also supported by work showing that individuals have less preference for alcohol products with health warnings (Jarvis and Pettigrew 2013). Hence, the lower preference for alcoholic beverages with health warnings may be manifested in the present study as a slower speed of consumption.

The observation that consumption speed did not differ between the pictorial and text-only conditions was surprising and contrary to prediction. In the previous study (Wigg and Stafford 2016), we found higher levels of fear in the pictorial versus text condition which made it seem probable to expect a slower consumption in the pictorial condition. This would also be consistent with the Extended Parallel Processing Model (EPPM, De Hoog et al. 2007) where health messages accompanied by threatening visuals have larger effects, as found in both alcohol (Slater et al. 2002) and tobacco research (Hammond et al. 2004; Schneider et al. 2012). However, it needs to be emphasised that those studies used different outcome measures from the study here, including perception of risk (alcohol) and intentions to reduce or quit (smoking). It could be therefore that the differences between pictorial and text-only health warnings are less important when measuring speed of consumption. Alternatively, it could be that the rate at which alcohol is consumed is not sufficiently sensitive to capture these differences and that adopting a different measure such as volume of consumption might be more appropriate. Connected to this, one study has found that the amount of alcohol consumed is sensitive when assessing the effectiveness of cognitive restraint training (Jones et al. 2011).

Given the aforementioned, the finding that pictorial health-warning labels led to lower acceptability ratings was not surprising; this is also consistent with work demonstrating that a cigarette’s brand appeal can be reduced in the presence of health-warning labels (Thrasher et al. 2007). More interestingly was the finding that despite a clear difference in consumption speed, there were no corresponding difference in acceptability between the text and control conditions, which suggests that many of the advantages of including a health warning are available in the absence of any strong reductions in product acceptability. These findings also extend a previous study where alcohol warnings related to cancer did not provoke overly negative reactions (Pettigrew et al. 2014).

The absence of a relation between protective drinking behaviour and consumption rate is somewhat surprising. Since one of the components of that measure specifically relates to the habitual practice of pacing drinks, it seemed reasonable to assume that this would relate to actual speed of consumption. Previous research using the protective behaviour instrument found it had good accuracy in predicting future alcohol consumption over a given time period (Ray et al. 2009), but we are not aware of any work that examined its possible link with episodic drinking rate. It therefore seems likely that protective behaviours relate more to longer periods of self-reported alcohol consumption.

Considering the wider implications of the present study, since the speed of alcohol consumption influences the level of intoxication and associated health risks (Moskowitz and Burns 1976; Bernosky‐Smith et al. 2012), the findings here have particular resonance; using such health-warning labels can help inform the public and could lead to safer drinking practices. These findings also have implications for wider alcohol work that has examined the drivers of consumption rate in terms of environment (e.g. music, Stafford and Dodd 2013) and sensory/behavioural changes (Higgs et al. 2008). The present study extends those areas of research to show that consumption rate is also sensitive to the information presented on the alcohol product.

In terms of study limitations, due to the fact that this was a female only study, we cannot be sure if the impact of health warnings would be the same for male participants. The rationale to use females was based on the likely confound of consumption rate differences between genders and also the difficulty in finding an alcohol beverage consumed widely by both males and females; the use of one gender is also consistent with previous consumption rate research, (Higgs et al. 2008; Stafford and Dodd 2013). Interestingly, one might speculate that the effects observed in the present study would be weaker for males, based on the fact that work has shown that in response to alcohol health-warning advertisements, females responded more positively than males (Slater et al. 2002).

In summary, we found that individuals consumed alcohol more slowly when it was presented with pictorial or text-only health warnings. This is the first study to demonstrate this outcome measure in the context of health-warning messages similar to those used in the tobacco industry. The findings here suggest that using health warnings on alcoholic beverages could be an effective strategy in changing attitudes and behaviour to one of our favourite but harmful drugs.
